# The effects of green and chemically-synthesized copper oxide nanoparticles on the production and gene expression of morphinan alkaloids in Oriental poppy

**DOI:** 10.1038/s41598-024-56709-8

**Published:** 2024-03-12

**Authors:** Iman khaldari, Mohammad Reza Naghavi, Elaheh Motamedi, Meisam Zargar

**Affiliations:** 1 Division of Biotechnology, Department of Agronomy and Plant Breeding, Agricultural and Natural Resources College, University of Tehran, Karaj, Iran; 2 Department of Nanotechnology, Agricultural Research, Education and Extension Organization (AREEO), Agricultural Biotechnology Research Institute of Iran (ABRII), Karaj, Iran; 3 Department of Agrobiotechnology, Agrarian Technological Institute, RUDN University, Moscow, Russia

**Keywords:** *Papaver orientale*, Copper oxide nanoparticles, Alkaloids, Elicitor, Gene expression, Biotechnology, Molecular biology, Plant sciences

## Abstract

Oriental poppy (*Papaver orientale* L.) belonging to the Papaveraceae family, has the capacity to synthesize a wide range of benzylisoquinoline alkaloids (BIAs). This experiment was conducted to investigate the effects of green and chemical copper oxide nanoparticles (CuO NPs) elicitors on oxidative stress and the BIAs biosynthesis pathway in the cell suspension culture of *P. orientale*. This research shows that both green and chemical CuO NPs at concentrations of 20 mg/L and 40 mg/L, induce oxidative stress in the cell suspension of *P. orientale* by increasing the production of H_2_O_2_ and the activity of antioxidant enzymes. The comparison of treatments revealed that utilizing a lower concentration of CuO NPs (20 mg/L) and extending the duration of cell suspension incubation (up to 48 h) play a more influential role in inducing the expression of the BIAs biosynthesis pathway genes (*PsWRKY*, *TYDC*, *SalSyn*, *SalR*, *SalAT*, *T6ODM*, *COR* and *CODM*) and increasing the production of morphinan alkaloids (thebaine, codeine, and morphine). The overarching results indicate that the concentration of CuO NPs and the duration of cell treatment have a more significant impact than the nature of CuO NPs in inducing oxidative stress and stimulating the expression of the BIAs pathway genes.

## Introduction

Until the arrival of iatrochemistry in the sixteenth century, plants were the main source of treatment and prevention of diseases^1^. The oldest evidence of the use of plants as drugs was found on a Sumerian clay slab approximately 5000 years ago. It includes 12 instructions for the preparation of drugs and refers to more than 250 different plants, the most well-known of which are henbane (*Hyoscyamus niger*), mandrake (*Mandragora sp*.), and opium poppy (*Papaver somniferum* L.)^1^. Today, the medicinal use of plants is based on the isolation of active compounds, which began with the isolation of morphine from the poppy in the early nineteenth century^2,3^.

The Papaveraceae family, belonging to the Ranunculales order, includes flowering and dicotyledonous plants and contains about 775 plant species^4^. The importance of the Papaveraceae family is related to its ability to synthesize a group of alkaloids called benzylisoquinoline (BIAs), comprising more than 2500 different nitrogen-containing compounds^5^. Although the *P. somniferum* L. is considered the most important species of the Papaveraceae family (the main natural source of morphinan alkaloids), the results of numerous research emphasize the ability of many species within the Papaver genus to synthesize plant active compounds, especially BIAs^6,7^. Orientale poppy (*Papaver orientale* L.), a tetraploid species (2n = 4x = 28), has a close genetic affinity with the Persian poppy (*Papaver bracteatum* Lindl.). It is naturally distributed in northwestern Iran and northeastern Turkey and it is considered as a rich source of thebaine and oripavine alkaloid^8,9^.

**Figure 1:**
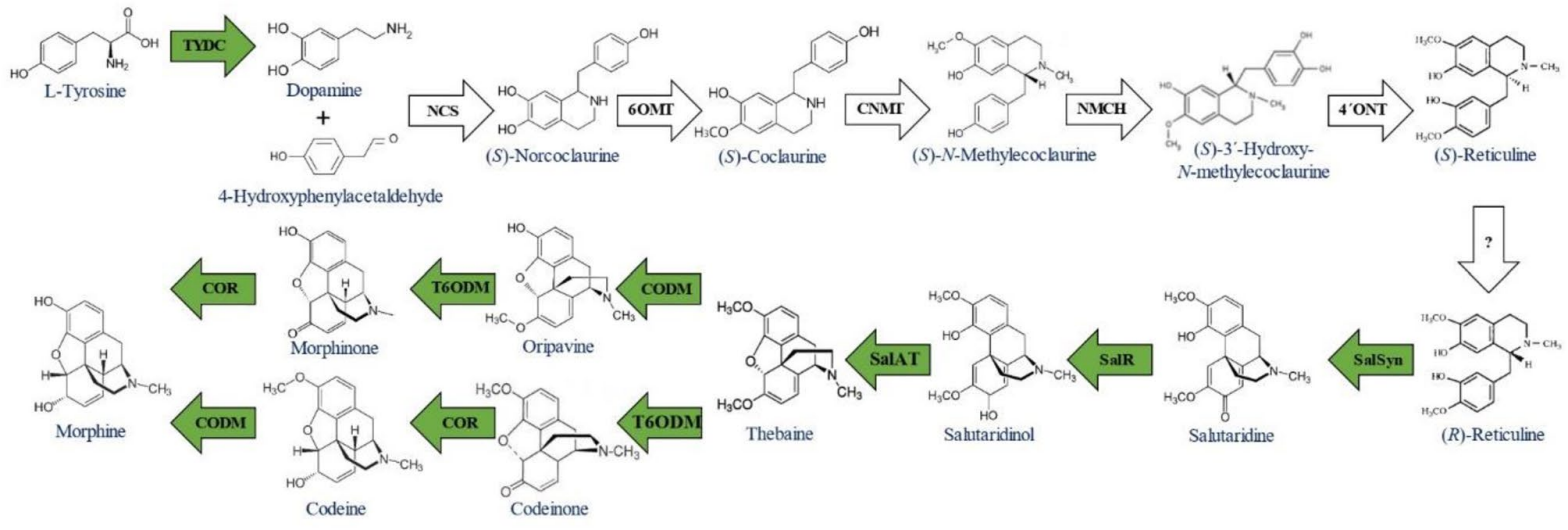
A part of BIAs biosynthetic pathway that ends in morphinan alkaloids. The arrows indicate the enzymes which are involved in alkaloids biosynthesis. The green arrows show the investigated genes. tyrosine decarboxylase (*TYDC*), norcoclaurine synthase (*NCS*), norcoclaurine 6-O-methyltransferase (*6OMT*), coclaurine N-methyltransferase (*CNMT*), N-methylcoclaurine (*NMCH*), 3-Hydroxyl-N-methylcoclaurine 4-Omethyltransferase 3-hydroxylase (*4'OMT*), salutaridine synthase (*SalSyn*), salutaridinereductase (*SalR*), salutaridinol 7-O-acetyltransferase (*SAT*), thebaine 6-O-demethylase (*T6ODM*), codeinonereductase (*COR*), codeine O-demethylase (*CODM*) Adapted from Beaudoin and Facchini^12^).

The BIAs comprise about 2500 known nitrogen-containing structures with special pharmacological importance. Among the most significant are morphine and codeine (narcotic analgesic), papaverine (muscle relaxant), sanguinarine (antibacterial agent) and berberine (cholesterol reducer)^10,11^. The biosynthesis pathway of BIAs in the Papaveraceae family is an ever longer synthetic pathway that begins with the formation and fusion of two L-tyrosine derivatives through the enzymatic process of TYDC, TyrAT, and NCS enzymes. Following, the main such as *SalR*, *SalSyn*, *SalAT*, *T6ODM*, *CODM* and *COR* direct the pathway towards the synthesis of morphinan alkaloids such as thebaine, oripavine, codeine and morphine^12^ (Fig. 1).

Previous research has confirmed the presence of 9% thebaine and 20% oripavine in dried latex obtained from *P. orientale* capsules^9^. As well as recent research has shown that *P. orientale* hairy root cultures are able to synthesize codeine, thebaine, and morphine^13^. Therefore, more research on the ability to synthesize morphinan alkaloids by *P. orientale* seems imperative.

**Figure 2:**
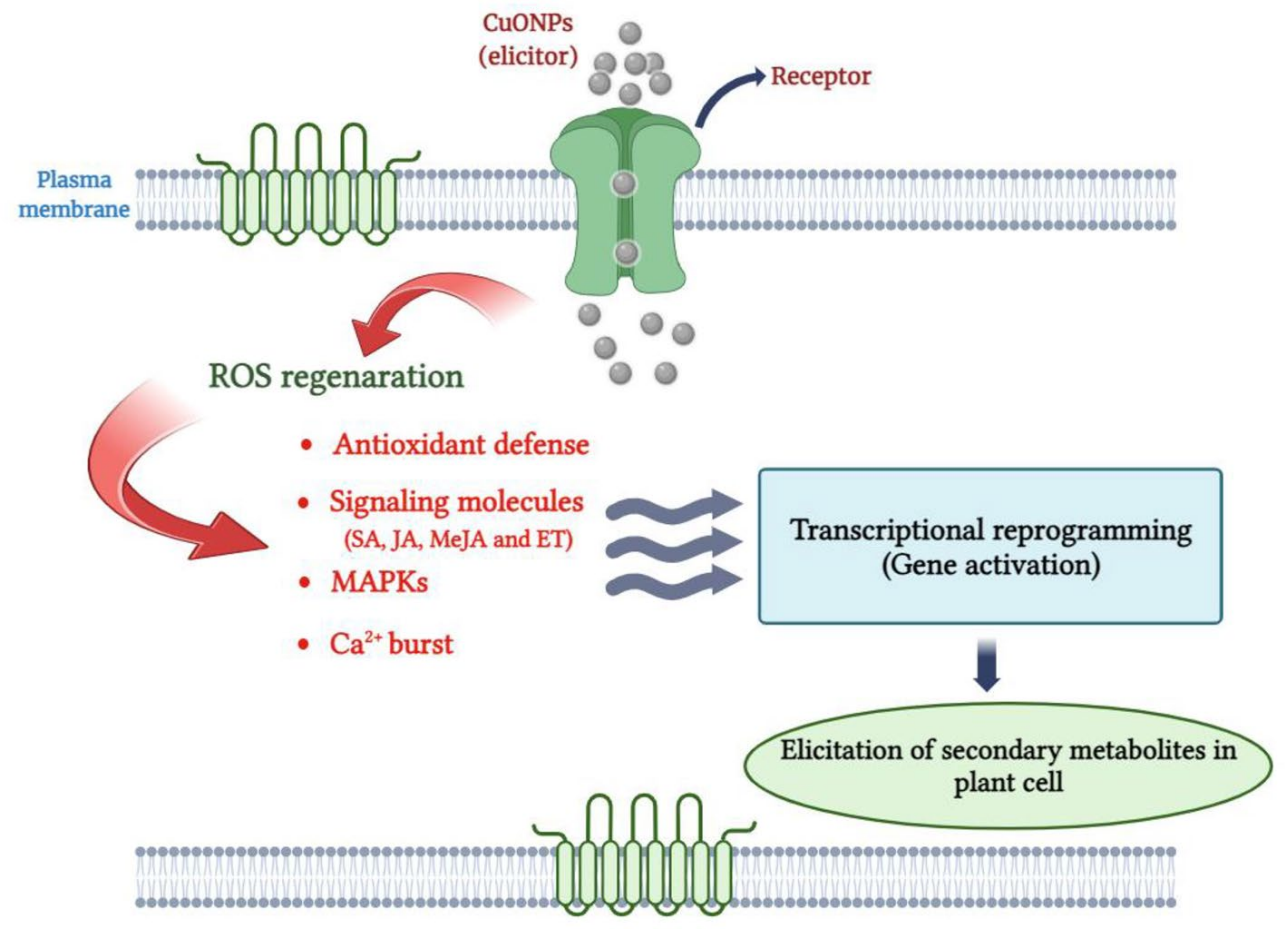
A schematic paradigm delineates the intricacies of CuO NPs exposure-induced biosynthetic cascades of secondary metabolites within the cellular milieu of plants. Consequent to the imposition of CuO NPs treatment, the interplay between nanomaterials and cellular components engenders the prolific generation of (ROS)^27^. The ROS elicited by NPs intricately mediates multifaceted responses, including the mediation of mitogen-activated protein kinase (MAPKs), Ca2 + burst^28^, modulation of signaling molecules, and fortification of antioxidant defense mechanisms^29^, thereby potentially orchestrating a nuanced modulation in the transcriptional dynamics governing secondary metabolism. In response to ROS exposure, the plant cellular apparatus promptly initiates a defensive response, carefully executed to prevent the harmful effects of ROS-induced damage.

In recent decades, different methods of plant tissue culture as well as the use of elicitors for the commercial production of plant secondary metabolites and the identification of cellular and molecular mechanisms involved in the production of these metabolites have been investigated^14^. In general, nanoparticles (NPs) are known as potentially toxic agents for plant cells. These nanoscale elicitors exert their toxic effects by generating reactive oxygen species (ROS), to which plants respond by activating the non‐enzymatic and enzymatic antioxidant defense mechanisms (Fig. 2). Also, the increase in the biosynthesis of secondary metabolites in plant cells has been proven as one important manifestation of the defense response against oxidative stress^15^. The effect of NPs on biochemical processes and the induction of oxidative stress in plant cells has been determined by several studies. For instance, the induction of oxidative stress in *Satureja khuzestanica* calli treated with Multi-walled carbon nanotubes (MWCNTs)^16^, the induction of catalase activity in *Stevia* *rebaudiana* L. calli treated with copper nanoparticles (Cu NPs)^17^ and effect of silver nanoparticles (Ag NPs) on antioxidant enzyme activity in *Caralluma* *tuberculate* cells^18^. Moreover, it seems that oxidative stress is related to signaling pathways leading to the synthesis of secondary metabolites^19^.

Among different elicitors, NPs are known as effective elicitors in the production of secondary metabolites due to their unique properties. Recently, various types of NPs have been used as new and effective elicitors in the laboratory cultivation conditions of different plant species^20^. Various metal nanoparticles, metal oxide nanoparticles, and carbon-based nanoparticles have been reported as effective NPs. These NPs include Ag NPs, Cu NPs, iron oxide nanoparticles (Fe_3_O_4_ NPs), zinc oxide nanoparticles (ZnO NPs), copper oxide nanoparticles (CuO NPs), carbon nanotubes and chitosan-based nanoparticles^21^.

Overall, all NPs synthesis protocols are divided into three main classes; physical, chemical, and biological (or green). The naming of each of these methods is based on the reducing agent source involved in the NPs synthesis process. In the physical method, a physical source, such as the electric current, serves as the electron source; in the chemical method, it is a chemical source, and in the biological method, it is a biomolecule or an organism^22,23^. It is noteworthy that, despite the potentially toxic effects of NPs on biological systems, some studies emphasize the milder toxic effects of green nanoparticles on plant cells^24,25^.

Although there is limited information about the upstream control mechanisms of biosynthesis pathways of secondary metabolites, it has been reported that the *PSWRKY* transcription factor plays an effective role in controlling the BIAs pathway through binding to the regulatory regions of the *TYDC*. In addition, it has been found that *PsWRKY* is induced by several stimuli such as cold, wound and salt stress, as well as methyl jasmonate and abscisic acid hormones^26^.

In this study, for the first time, to achieve the best conditions for callus formation and growth, we investigated the ability of callus formation of different *P. orientale* explants under the influence of different plant growth regulators (PGRs). Additionally, we investigated the effect of green and chemical CuO NPs on oxidative stress induction, expression of some BIAs pathway genes and morphinan production to establish the best conditions for the biosynthesis of these important secondary metabolites. *P. orientale* cell suspension cultures were used for the experiments due to their moderate uniformity, making them easier to use and study the effect of NPs.

## Materials and methods

### Plant materials

The seeds of *P. orientale* were provided from the gene bank of IPK Gatersleben. Normal seeds were sterilized with 70% ethanol for 1 min and then with a 1% sodium hypochlorite solution for 10 min, then rinsed four times in sterile distilled water. These sterilized seeds were cultured on a basal medium consisting of Murashige and Skoog (MS) salts and vitamins and 30 g/L sucrose solidified using 7 g/L agar. The pH of the medium was adjusted to 5.8 before adding agar, and the medium was sterilized by autoclaving at 15 psi and 120 ℃ for 20 min. The seed germination was performed in a growth chamber at 23 ℃ under a 16/8 h (light/dark) photoperiod, with a light intensity of 1500 lx. After 45 days, segments of roots, collars, leaves and petioles were prepared for callus induction.

### Callus induction

Calli were inducted on MS medium containing 7 g/L agar, 30 g/L sucrose and a combination of 6-benzylamino purine (BAP) at two levels of 0.5 and 1 mg/L with naphthaleneacetic acid (NAA) or 2,4-Dichlorophenoxyacetic acid (2,4-D) at two levels of 1 and 2 mg/L, serving as plant growth regulators, at 23 ℃ under dark condition. The generated calli were regularly sub-cultured every 21 days.

### Cell suspension production

**Figure 3:**
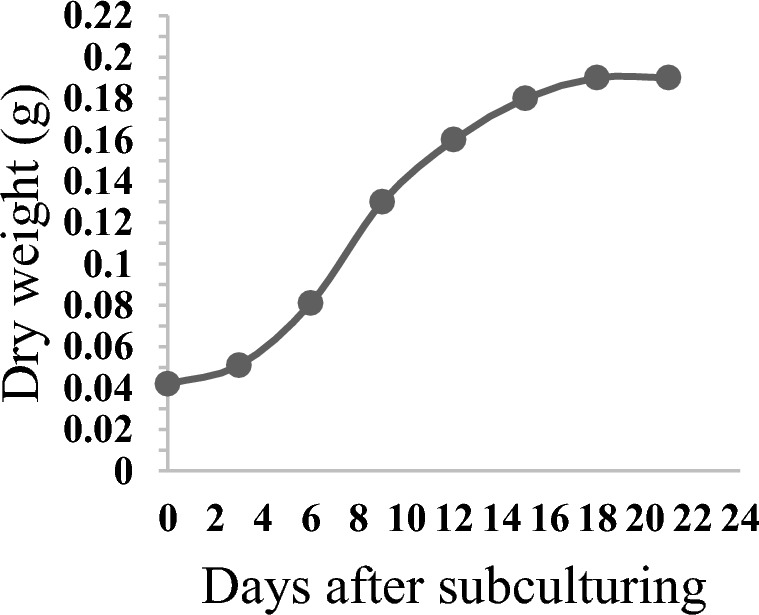
Growth curve of *P. orientale* cell suspension.

To prepare cell suspension culture, 0.5 g of callus obtained from the root explant was transferred into 50 mL of MS medium supplemented with 1 mg/L BAP, 2 mg/L NAA and 30 g/L sucrose. Afterward, the culture media was incubated on a rotary shaker at 120 rpm and 23 ℃ under dark conditions. The cell suspension culture medium was substituted with fresh culture medium every 21 days. In the third sub-culture, the cell suspension growth curve was prepared by assessing the dry weight of cells every three days. To determine the culture’s dry weight, the cells were separated by filtering the culture medium and then washed with distilled water. Finally, to obtain the biomass dry weight, the harvested cells were dried at 50 ℃ in a hot air oven^30^. Based on the drawn growth curve (Fig. 3), before the growth of the cells touched the stationary stage, the CuO NPs treatments were carried out (12 days after the last sub-culture).

### Nanoelicitors treatments

CuO NPs used in this research were synthesized in our previous study and their quality measurement and toxicity level on plant cells were subsequently investigated^25^. To apply the CuO NPs treatments, the green and chemical CuO NPs powder was added to the MS basal medium and then autoclaved. Moreover, for better dispersion of NPs, the autoclaved culture medium was placed in an ultrasonic bath for 30 min. Finally, cell suspension cultures were fortified at CuO NPs with two concentrations of 20 and 40 mg/L, and the treated cultures were incubated for 24 and 48 h. For each treatment, three replicates were evaluated. After incubation, the treated cells were detached from the culture medium by filtration. Subsequently, the biomass was divided into three parts, and these parts were frozen in liquid nitrogen and stored at -80 ℃ for total RNA extraction, alkaloids measurement, and biochemical analysis.

### RNA extraction and quantitative real-time PCR analysis

Treated cells (100 mg) were prepared for RNA extraction. The cells were powdered using a cold mortar and pestle in liquid nitrogen. RNA extraction was carried out according to the manufacturer's instructions (Bio-Equip) using p-BIOZOL. RNA quality was assessed by NanoDrop ND-1000 spectrophotometer (Thermo Fisher Scientific Inc, USA). Afterward, 2 μg of RNA was treated with DNase I (Fermentas, USA), and the first strand of cDNA was performed using the cDNA synthesis kit (Parstous, Iran) according to the manufacturer's instructions.

The coding sequences of *PsWRKY*, *TYDC*, *SalSyn*, *SalR*, *SalAT*, *CODM*, *T6ODM*, *COR* and *Elf1α* were obtained from the National Center for Biotechnology Information (NCBI). Primer design was conducted using Primer 3 and PrimerQuest and then Oligo Analyzer and Primer Blast were employed to check the quality factors of the synthesized primers. The primer sequences (5'-3') are listed in Table 1.

**Table 1:** Primer list for target genes^12,26^ used in qRT-PCR.

Primer	Accession no	Sequence
*PsWRKY*	JQ775582	F-TGTTATTCGGATCGGACTGT
		R-CCATATCATAAAACCAAGGACTTAAGG
*TYDC*	EU882987	F-AACCCACTAGACCCTGATGA
		R-GACCTGGCTTCTAACTGGATAAC
*SalSyn*	EF451150	F-CGGCGAGCAGGAAATTCAAG
		R-GGCAGCTCTTTCAAATCTACAAC
*SalR*	DQ316261	F-TGGAAGTCCGTGATGAAATCC
		R-GCTGGTAAGAACGCCGAAAC
*SalAT*	KR260912	F-TGGAAGTCCGTGATGAAATCC
		R-GCTGGTAAGAACGCCGAAAC
*CODM*	GQ500141	F-TTGTGCTTAAATTTCGTGGATGAC
		R-TGATTACATCACTTGACCCAAACAG
*T6ODM*	GQ500139	F-AAAACTCCCAGTGCCTCTCA
		R-ACCCTTAATCTCGGCTGCTT
*COR*	FJ624147	F-TTGATTGGGAACTAACGGCAGAAG
		R-TGAAAGGTCCAGTCGGTGATAACA
*Elf1a*	KF033667.1	F-AGATGATTCCAACCAAGCCCA
		R-CCTTGATGACACCAACAGCAACT

To investigate the relative expression of genes, qRT-PCR was conducted using 2X SYBR Green master mix (Parstous, Iran) in a 20 μL total volume containing 4 μL diluted cDNA, 0.5 μL of each primer and 10 μL 2X SYBR Green master mix. Real-time quantitative PCR was carried out at 5 min pre-denaturation at 94 ℃, 1 cycle, followed by 40 cycles, 15 s denaturation at 94 ℃; 30 s annealing temperature dedicated for each primer and 30s extension at 72 ℃. qRT-PCR reactions with tree technical replications.

were run in Rotor-Gene Q (Qiagen, Germany) to obtain PCR efficiencies, Ct values, and melting curves. Elongation factor 1 alpha (*Elf1α*) was used as an internal reference gene^31^. Relative gene expression was calculated using the REST 2009 software which uses ${\text{Ratio}}= \frac{{(\mathrm{Efficiency\, of\, target\, gene})}^{\Delta \mathrm{cp\, of\, target\, gene}}}{{(\mathrm{Efficiecy\, of\, reference\, gene})}^{\Delta \mathrm{cp\, reference\, gene}}}$ ^32^.

### Alkaloids measurement

The second portion of the treated cells was freeze-dried for alkaloid extraction. The freeze-dried cells were then powdered using mortar and pestle. One mL of methanol (99%) was added to 100 mg of the powdered cells and homogenized in a sonicator at 4 ℃ for 60 min in 2 mL tubes. Afterward, the homogenized cells were then kept at 4 ℃ for 12 h. Subsequently, the tubes were vortexed for 2 min and then centrifuged at 12000g for 10 min, and the supernatant was transferred to new tubes for alkaloids measurement^33^.

**Figure 4:**
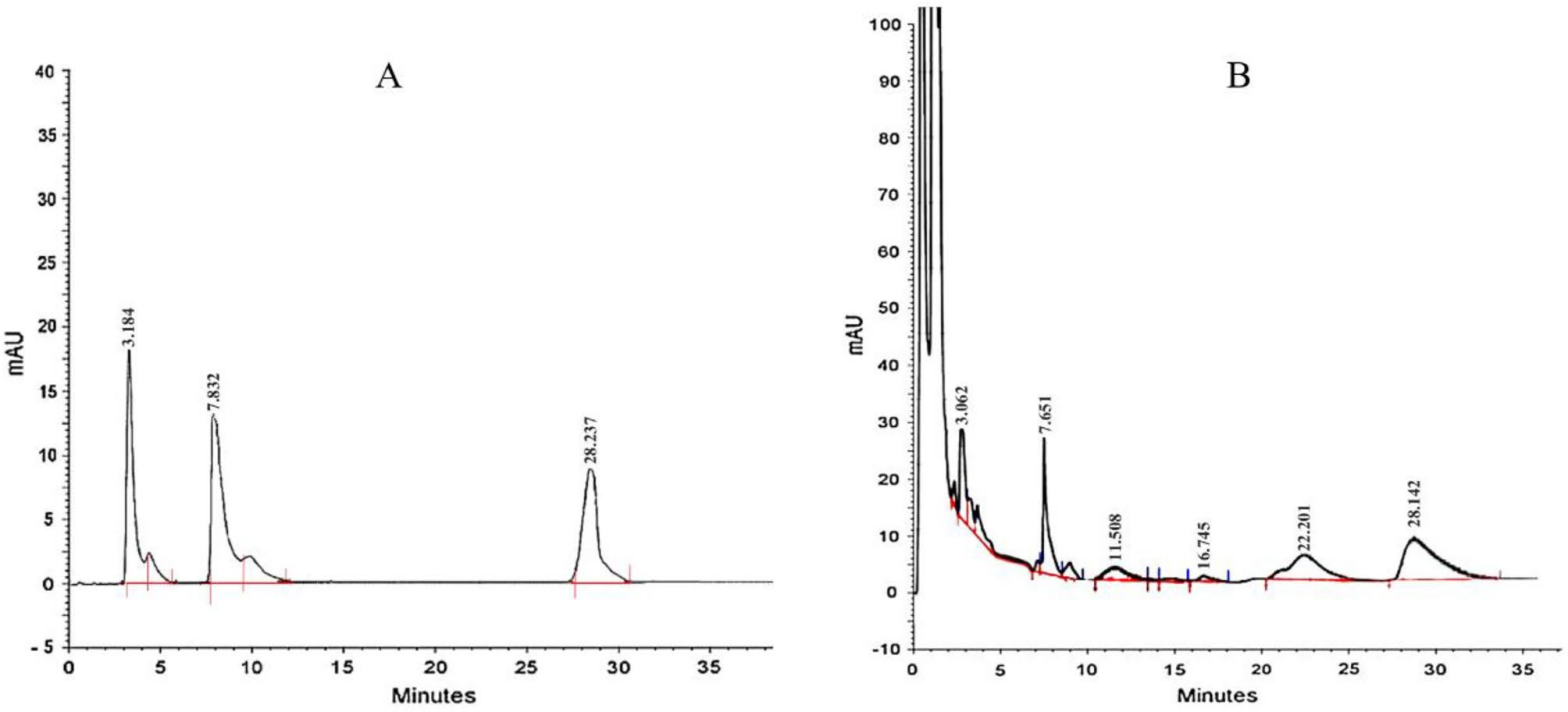
High performance liquid chromatography for standard of morphine, codeine, and thebaine (left peak, middle peak and right peak respectively) (**A**) and extract of *P. orientale* cell suspension elicited by 20 mg/L green CuO NPs at 48 h (**B**).

High-performance liquid chromatography (HPLC) (Knauer, PLATINblue, Germany) was used to determine metabolites. The samples were filtered using a syringe filter (0.22 μm) and 20 μL of each sample was injected into the HPLC nucleosil C18 column (250 mm × 4.6 mm, 5μm). The mobile phase consisted of 0.02 M KH2PO4: Acetonitrile (90:10 v/v) and the flow rate was 1 mL/min. The process was carried out at 25 ℃ and a UV detector with a wavelength of 254 nm was used. The experiment was repeated three times for each sample. Standard materials were acquired from Sigma Aldrich Company (Sigma, USA) to obtain calibration curve for thebaine, codeine and morphine (Fig. 4).

### Determination of hydrogen peroxide (H_2_O_2_) content

To determine the H_2_O_2_ content, 500 mg of treated cells were ground in an ice bath with 5 mL of trichloroacetic acid (TCA, 0.1% w/v). The homogenate was centrifuged for 15 min at 12,000 rpm and then 0.5 mL of 10 mM potassium phosphate buffer (pH 7.0) and 1 mL of 1 M KI were added to 0.5 mL of the supernatant. The absorbance of the samples was measured at 390 nm. To calculate the H_2_O_2_ content in the samples, a standard calibration curve was obtained from different concentrations of H_2_O_2_ and expressed in µmol/g FW^34^.

### Enzyme extraction and assay

Total soluble protein content was determined based on the Bradford method^35^. 500 mg of treated cells were ground in liquid nitrogen, and enzyme extraction was carried out at 4°C. The crushed cells were homogenized in the extraction buffer (Tris–HCl, pH 7.8, containing 10% glycerol). The extracts were then centrifuged at 15,000 rpm for 15 min at 4°C. The supernatant was then used for the catalase (CAT), guaiacol peroxidase (GPX), and ascorbate peroxidase (APX) assays.

CAT activity was determined by monitoring the H_2_O_2_ disappearance^36^. The reaction mixture contained 3 mL of phosphate buffer (pH 7.0), 5 µL of 30% H_2_O_2_ and 50 µL of crude enzyme extract. The CAT activity was determined based on a decrease in absorbance at 240 nm for 1 min and defined as µmol of H_2_O_2_ decomposed/ (min mg protein).

GPX activity was measured by adding 50 µL of crude enzyme to the reaction mixture. The reaction mixture consisted of 3 µL of guaiacol and 10 µL of 30% H_2_O_2_ in 3 mL of sodium phosphate buffer, pH 7.0^37^. Changes in absorbance at 470 nm were recorded for 1 min, and the activity of GPX was expressed in µmol of guaiacol oxidized / (min mg protein).

The reaction mixture consisted of 100 µL of enzyme extract, 600 µL of 0.1 mM EDTA in 1.5 mL of 0.05 M potassium phosphate buffer (pH 7.0), and 400 µL of 0.5 mM ascorbic acid. The reaction started with the addition of 400 µL of 30% H_2_O_2_. APX activity was determined by measuring the decrease in ascorbate content at 290 nm for 4 min^38^. The activity of APX was expressed in µmol of ascorbate oxidized/ (min mg protein).

### Statistical analysis

The results of the experiments were statistically analyzed using SAS 9.3.1 Portable. The recorded data were processed by analysis of variance (ANOVA) on the basis of Completely Randomized Design (CRD) with three replications (callus induction experiment was designed as factorial experiment). The significance of the difference between treatment means was carried out by Duncan’s Multiple Range Test (DMRT) at *p* < 0.05 level. The results were presented in the form of a combination of treatments, rather than separately or individually. In the pursuit of identifying optimal treatments for the induction of both gene expression and morphinan alkaloid accumulation, a hierarchical cluster analysis (HCA) was implemented. This analytical approach, complemented by heatmap visualization, was executed utilizing the ClustVis web-tool^39^. The calculation of correlations between gene expression data and morphinan alkaloid values was conducted through the application of the corrplot function within the RStudio environment (R 4.3.2)^40^.

### Author statement

Experimental research and field studies on seeds complied with relevant institutional, national, and international guidelines and legislation.

## Results

### Callus induction in different explant

**Figure 5:**
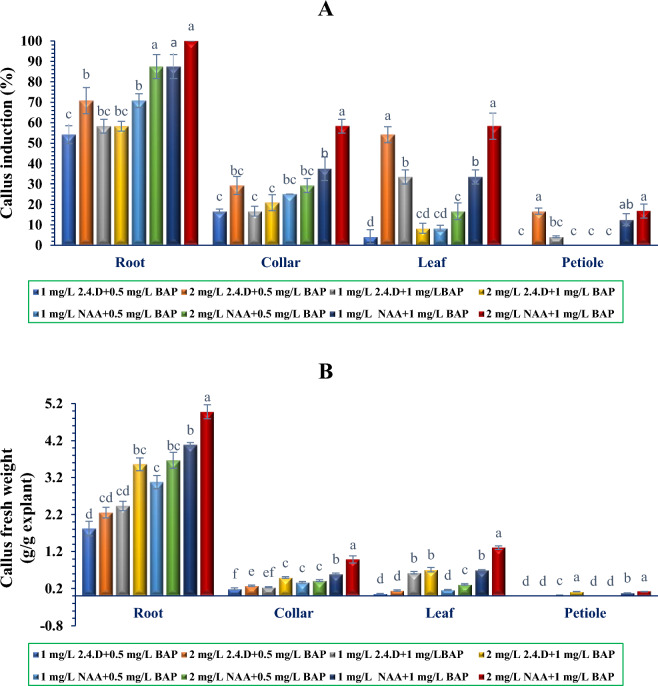
Comparative changes in the callus induction percentage (**A**) and biomass production (**B**) in *P. orientale* explants treated with different concentrations of PGRs. Error bars represent ± SE. Different letters above the columns represent statistically significant differences at *P* < 0.05 (Duncan’s multiple range test).

Firstly, four different *P. orientale* tissues, roots, collars, leaves and petioles were employed as explants for callus induction. Among these four explants, roots showed the highest percentage of callus formation, ranging from 54.2% to 100% across the eight media supplemented with different concentrations of PGRs. Subsequently, collars exhibited callus formation ranging from 16.7% to 58.3%, while leaves showed a range of 4.2% to 58.3% and petioles explants showed the lowest percentage of callus formation from 0% to 16.7% (Fig. 5A). The fresh weights of callus were recorded after two subcultures to determine the biomass accumulation. The maximum callus fresh weights, were achieved from root explants ranging from 3.64 to 9.96 g/g explant. The mentioned trait was measured from 0.12 to 2.62 g/g explant in leaves and from 0.38 to 1.97 g/g explant in collar. It is worth mentioning that the lowest amount of callus production was observed in the petiole explants, ranging from 0 to 0.25 g/g explant (Fig. 5B) To assess the effects of plant growth regulators on *P. orientale* callus formation and callus fresh weight, combinations of 2.4-D and NAA (auxins) with BAP (cytokinin) were employed. The results demonstrated successful induction of callus formation in all four explants. The comparison of different plant growth regulator compositions revealed that the combination of the highest concentrations of 2.4-D or NAA (2 mg/L) with BAP (1 mg/L) was more effective in inducting callus and increasing callus fresh weight than the combination of the lowest concentration of 2.4-D or NAA (1 mg/L) with BAP (0.5 mg/L). Also, other compounds demonstrated an intermediate effect on the two mentioned traits. Generally, at an equal concentration, NAA was a better auxin than 2.4-D for callus induction and callus fresh weight. Based on these observations, the calli obtained from the roots and the combination of NAA (2 mg/L) with BAP (1 mg/L) were selected as the most favorable combination for the continuation of the experiment (sub-culturing and production of cell suspension).

### ***H***_***2***_***O***_***2***_*** content and enzymatic activity***

**Figure 6:**
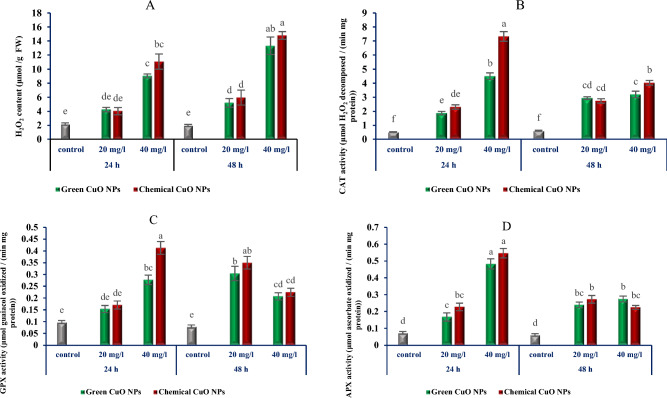
The effects of different concentrations of green and chemical CuO NPs at different times (24 & 48 h) on H_2_O_2_ production (**a**) and the activity of antioxidant enzymes CAT (**b**), GPX (**c**) and APX (**d**) in cell suspension culture of *P. orientale.* Values are means of three replicates. Error bars represent ± SE. Different letters above the columns represent statistically significant differences at *P* < 0.05 (Duncan’s multiple range test).

The application of 20 mg/L CuO NPs increased the H_2_O_2_ generation compared to controls at both 24 and 48 h. At the concentration of 40 mg/L CuO NPs, more H_2_O_2_ production was observed at 24 h, which continued to increase after 48 h. Therefore, the highest amount of H_2_O_2_ was recorded at a concentration of 40 mg/L CuO NPs at 48 h. However, no significant changes were observed in the amount of H_2_O_2_ production between the treatments with green and chemical CuO NPs (Fig. 6A).

CAT activity in the different treatments exhibited varying increases (Fig. 6B). The activity of CAT at the concentration of 20 mg/L CuO NPs at both 24 and 48 h was higher than the control treatment at the same rate. CAT activity at the concentration of 40 mg/L CuO NPs at 24 h was dramatically higher than the control but it decreased with increasing treatment time (at 48 h). Notably, at the concentration of 40 mg/L CuO NPs, the superiority of chemical CuO NPs in increasing CAT activity was observed.

In response to CuO NPs elicitors, a regular alteration in the activity of GPX was observed at both concentrations. GPX activity did not exhibit a significant change at 20 mg/L at 24 h, but an increase in incubation time up to 48 h resulted in a significant rise in GPX activity. At the concentration of 40 mg/L CuO NPs, the GPX activity pattern was similar to the CAT activity, with an initial increase at 24 h followed by a subsequent decline at 48 h (Fig. 6C).

It was observed that APX activity at the lowest level of CuO NPs (20 mg/L) showed a significant increase compared to the control condition (Fig. 6D). Further, observations revealed that increasing the concentration of CuO NPs up to 40 mg/L resulted in a noteworthy increase in APX activity. Therefore, increasing the concentration of CuO NPs up to 40 mg/L initially (24 h) led to a remarkable increase in APX activity. However, with the increasing incubation time of cell suspension cultures (48 h), the APX activity decreased.

Despite the variation in H_2_O_2_ generation rate and CAT, GPX and APX activity in different treatments, no significant difference was observed in similar treatments of green and chemical CuO NPs.

### Relative expression of BIAs pathway genes

**Figure 7:**
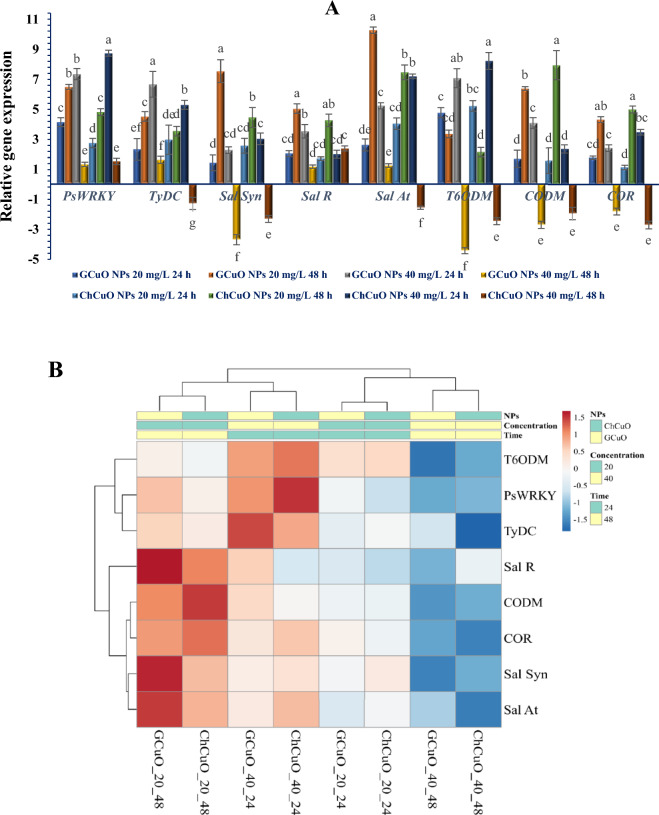
The effects of different concentrations (20 & 40 mg/L) of green and chemical CuO NPs at different times (24 & 48 h) on relative expression of some BIAs pathway genes in cell suspension culture of *P. orientale.* Values are means of three replicates. Error bars represent ± SE. Different letters above the columns represent statistically significant differences at *P* < 0.05 (Duncan’s multiple range test) (**A**). Heat-map clustered for all CuO NPs treatments (blue and red colors indicate the lowest and highest association degrees between each pairwise comparison, respectively.). Both rows and columns are clustered using maximum distance and average linkage (**B**).

The relative expression of genes in cell suspension treated with different concentrations of CuO NPs is reported in Fig. 7A. The expression of *PsWRKY* and *TYDC* increased at the concentration of 20 mg/L CuO NPs at 24 h and a higher relative expression was observed at 48 h. However, at the concentration of 40 mg/L CuO NPs, the highest expression rate of *PsWRKY* and *TYDC* was observed after 24 h and with increasing time up to 48 h, the lowest level of transcript accumulation of both genes was observed (Fig. 7A).

The relative expression of *SalSyn*, *SalR*, *SalAT*, *COR* and *CODM* exhibited a relatively similar pattern. Therefore, the lowest level of CuO NPs elicitors used (20 mg/L) caused a slight up-regulation of genes at 24 h and after 48 h, the maximum level of transcripts accumulation was detected. In contrast the expression rate in the treatments containing the highest level of CuO NPs (40 mg/L) showed a different trend. Thus, the relative expression observed at 24 h decreased significantly at 48 h (in most genes with negative relative expression) (Fig. 7A).

An impressive accumulation of *T6ODM* transcripts was observed in both concentrations of CuO NPs (20 and 40 mg/L) at 24 h. However, with an increase in the incubation time up to 48 h, a downward trend of *T6ODM* gene expression was detectable in both concentrations of CuO NPs (Fig. 7A).

Upon comparing corresponding treatments, various conditions emerged, including instances of the superiority of green CuO NPs, the superiority of chemical CuO NPs, or a lack of significant difference between the two CuO NPs. However, the overall outcomes suggest a uniformity in the impact on the expression of BIAs biosynthesis genes between green and chemical CuO NPs. The heat-map clustered output indicates that treatments involving green and chemical CuO NPs, characterized by identical concentrations and durations, exhibit no significant differences. As a corollary, these treatments are unequivocally consigned to the same categorical classification. As delineated by the clustered heat map, treatments characterized by a concentration of 20 mg/L over a 48 h duration and 40 mg/L over 24 h exhibited the most pronounced impact on the accumulation of transcripts of the genes associated with the BIAs pathway and forming a distinct cluster (Fig. 7B). In contradistinction, the influence of alternative treatments on gene expression witnessed a notable reduction, thereby giving rise to the formation of a discrete secondary cluster (Fig. 7B).

### Measurement of morphinan alkaloids

**Figure 8:**
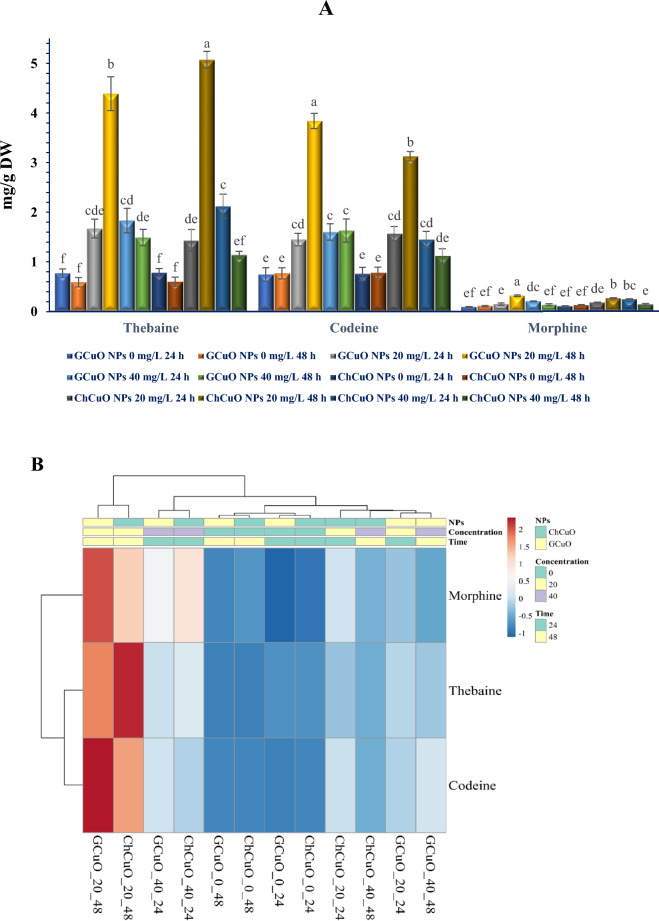
The effects of different concentrations (0, 20 & 40 mg/L) of green and chemical CuO NPs at different times (24 & 48 h) on morphinan alkaloids production (mg/g DW) in cell suspension culture of *P. orientale.* Values are means of three replicates. Error bars represent ± SE. Different letters above the columns represent statistically significant differences at *P* < 0.05 (Duncan’s multiple range test) (**A**). Heat-map clustered for all CuO NPs treatments (blue and red colors indicate the lowest and highest association degrees between each pairwise comparison, respectively.). Both rows and columns are clustered using maximum distance and average linkage (**B**).

To explore the impact of CuO NPs on the biosynthesis of morphinan alkaloids (including codeine, morphine, and thebaine) in the suspension cells of oriental poppy, HPLC was employed. The results show that although the biosynthesis of morphinan alkaloids is influenced by CuO NPs, the effect of some treatments on stimulating the accumulation of these alkaloids is far more significant. According to the results of the HPLC analysis, the application of 20 and 40 mg/L CuO NPs significantly increased the synthesis and accumulation of morphinan alkaloids compared to untreated controls. The highest thebaine accumulation in *P. orientale* cell suspension (5.07 mg/g DW) was observed when the lowest concentration of CuO NPs (20 mg/L) was applied at 48 h, while all other treatments showed the synthesis of thebaine up to 2.12 mg/g DW at their best performance (Fig. 8A). The results revealed that the maximum amount of codeine (3.84 mg/g DW) was obtained for the CuO NPs treatment at 20 mg/L after 48 h. Other treatments enriched with CuO NPs showed a lower amount of codeine, with the best treatment not exceeding 1.63 mg/g DW (Fig. 8A).

Morphine production in *P. orientale* cell suspension culture increased by adding CuO NPs so that the highest amount of morphine was observed in the treatment of 20 mg/L CuO NPs at 48 (up to 0.32 mg/g DW) and 40 mg/L CuO NPs at 24 h (up to 0.24 mg/g DW) respectively (Fig. 8A).

In a more comprehensive analysis, the cell suspension cultures treated with CuO NPs at a concentration of 20 mg/L showed increased accumulation of morphinan alkaloids with incubation time increasing from 0 to 24 h and from 24 to 48 h. However, cell cultures treated with a concentration of 40 mg/L of CuO NPs exhibited a significant decrease in the mentioned alkaloids with increasing time from 24 to 48 h (Fig. 8A).

The quantitative analysis of morphinan alkaloids showed that in *P. orientale* cell suspension cultures enriched with CuO NPs, thebaine and codeine were synthesized to a greater extent, respectively, while the amount of synthesized morphine was much less (Fig. 8A).

Similar to the outcomes observed in the induction of the BIAs biosynthesis pathway at the transcriptional level, the nature of the synthesis of CuO NPs (green or chemical) does not significantly affect the on the enhancement of morphinan quantities. In such a way that, in the results of the clustering analysis, green and chemical CuO NPs treatments with identical concentrations and incubation times are grouped together. The clustering analysis of treatments reveals that CuO NPs treatments with a concentration of 20 mg/L and a duration of 48 h exhibit the highest efficacy in the biosynthesis of morphinan, forming a distinct group. In contrast, treatments with significantly weaker performance are grouped into another distinct cluster (Fig. 8B).

### Correlation between relative gene expression and morphinan alkaloids quantities

**Figure 9:**
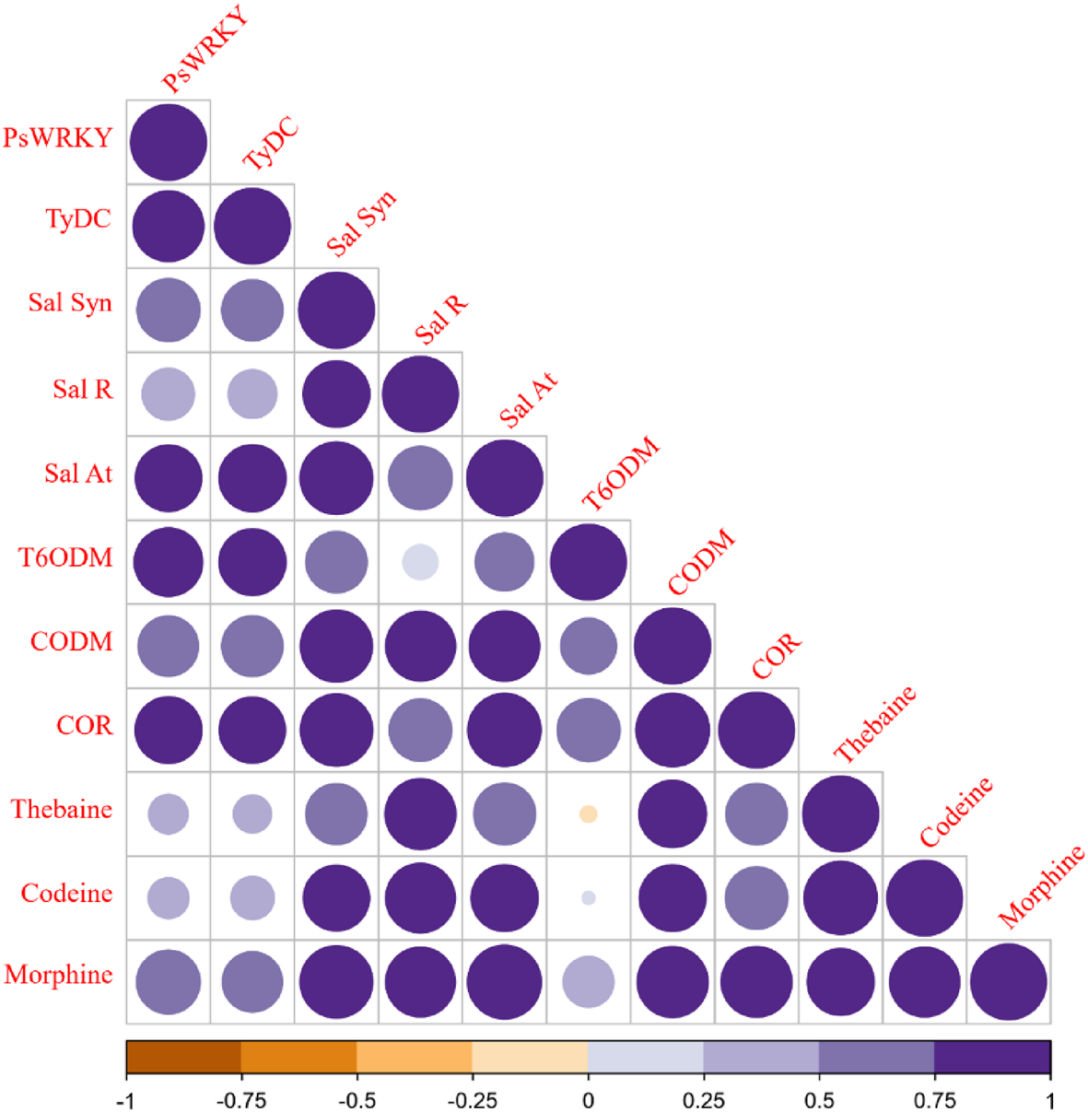
The corrplot-based graph to visually decipher the possible pairwise correlations among the relative gene expression data of some BIAs pathway genes and quantities of morphine, codeine and thebaine. The size of each circle and the shading volume of the colors are equivalent to the association degrees between each pairwise comparison.

To gain further insights into morphinan biosynthesis, we computed correlations between the expression levels of eight selected genes and the quantities of thebaine, codeine, and morphine (Fig. 9). Among the examined genes, the most notable correlation was identified between the expression of *CODM* and *COR* (*ρ* = 0.94, *P* < 0.01), *SalAT* and *COR* (*ρ* = 0.93, *P* < 0.01), *SalSyn* and *SalAT* (*ρ* = 0.92, *P* < 0.05), and *SalSyn* and *COR* (*ρ* = 0.91, *P* < 0.01), respectively. Additionally, the strongest correlation between the relative expression of the BIAs pathway genes and the biosynthesis of morphinan alkaloids was observed between morphine and *SalAT* (*ρ* = 0.95, *P* < 0.05) and morphine and *SalSyn* (*ρ* = 0.92, *P* < 0.01), respectively. Lastly, the highest correlation within the morphinan group was noted between thebaine and codeine (*ρ* = 0.94, *P* < 0.05) (Fig. 9).

## Discussion

In the commercial production of valuable plant secondary metabolites under tissue culture conditions, it is not only important to stimulate the production of these compounds, but it is also essential to optimize the conditions to achieve the desired amount of biomass. In this study, the observed differential callus induction was caused by the use of different explants, as well as various types and concentrations of cytokinins and auxins. Cytokinins and auxins are widely used PGRs in plant tissue culture, and are generally used in combination^41^. Previous studies have reported that the MS basal medium supplemented with 0.5 mg/L BAP and 1 mg/L NAA is the optimal medium for callus induction in *P. orientale*^42,43^, and *P. bracteatum*^44^. The results of our research also demonstrated the suitability of MS medium enriched with 0.5 mg/L BAP and 1 mg/L NAA for callus formation (up to 70.5% in root explants). However, we demonstrated that increasing of BAP and NAA concentrations up to 1 mg/L BAP and 2 mg/L NAA led to elevated callus induction (up to 100% in root explants) and callus fresh weight.

It appears that the augmentation of auxin and cytokinin in tissue culture conditions is associated with an increase in callogenesis and biomass. Therefore, some studies have reported that increasing the levels of NAA and BAP led to an increase in callus formation and biomass accumulation in *Gymnema* *sylvestre*^45^, *Digitalis lanata*^11^ and *Ocimum basilicum*^46^. The current research results also demonstrate that increasing the levels of each of the PGRs used leads to a higher percentage of callogenesis and biomass production.

Among PGRs, auxins play an important role in callus formation. Interestingly different types of auxins have different effects on callus induction^47^. In this research, it was evidently observed that the effect of NAA on callus induction and callus biomass formation was significantly higher than the effect of 2.4-D.

The root and petiole explants exhibited the highest (100%) and lowest (0%) amounts of callus induction, respectively. Given that different explants possess varying amounts of endogenous PGRs, and the role of the explant can influence callus induction^48^, it seems the polar transfer of auxin from the shoot to the root and the accumulation of auxin in the root^49,50^ can be effective factors in the induction of high callus in root tissue and the resulting high callus fresh weight.

Oxidative stress manifests in plant cells as a result of various adverse environmental conditions, including the presence of heavy metals or nanoparticles. Plant cells utilize a range of mechanisms to mitigate the hazards induced by ROS resulting from oxidative stress. The paramount strategies involve upregulating the activity of antioxidant enzymes and enhancing the biosynthesis and accumulation of secondary metabolites^51^. Therefore, comprehending the mechanism of stress induction and the cellular processes that lead to the production of secondary metabolites can enhance the efficiency of synthesizing valuable plant metabolites under tissue culture conditions.

Studying the production of Reactive Oxygen Species (ROS) and assessing the activity of both enzymatic and non-enzymatic antioxidant defense systems in cells are considered effective methods for understanding the occurrence of oxidative stress resulting from stress-inducing factors^52^. The generation of ROS, particularly H_2_O_2,_ in plant calli is known as oxidative stress, which triggers the activation of antioxidant defense systems in response to different types of stress conditions^53^. Several reports indicate an increase in the activity of antioxidant enzymes, a crucial component of the protective system in plant cells combating oxidative stress, under the treatment of plant cells with different NPs. For instance, increasing the activity of CAT in wheat seedlings treated with CuO NPs and ZnO NPs^54^, inducing the activity of CAT, APX, GPX and superoxide dismutase (SOD) and increasing the expression of related genes to oxidative stress in *Arabidopsis thaliana* treated with CuO NPs^55^. Most studies on the toxicity of NPs have focused on whole plant systems or seed germination. In contrast, interactions between NPs and plant cells in tissue and cell culture conditions have been somewhat neglected. However, a previous report^16^ showed an increase in the activity of antioxidant enzymes in *S. khuzestanica* calli treated with MWCNTs. According to our research results, the concentrations of 20 and 40 mg/L of both green and chemical CuO NPs increase the H_2_O_2_ content and the activity of CAT, APX and GPX in *P. orientale* cell suspension compared to the control. Therefore, it seems that CuO NPs are effective in inducing oxidative stress in *P. orientale* cell suspension. Despite numerous reports indicating the effect of NPs on increasing the activity of antioxidant enzymes, some reports point to a decrease in the activity of these enzymes in plant cells, tissues and organs treated with NPs. For instance, there are reports of the reduction in the activity of APX and GPX in *Lemna minor* seedlings treated with titanium oxide nanoparticles (TiO_2_ NPs)^56^. Additionally, the effect of TiO_2_ NPs on reducing the activity of CAT, GPX, and SOD in *Vicia faba* seedlings was observed^57^. According to the results, the production of H_2_O_2_ increased exponentially with the rise in the concentration of CuO NPs and the incubation time of the cells. However, despite the significant increase in antioxidant enzymes activity at the concentration of 40 mg/L CuO NPs at 24 h, increasing the incubation time of the *P. orientale* cell suspension up to 48 h with the highest concentration of CuO NPs used (40 mg/L) dramatically decreased the activity of CAT, APX and GPX.

It appears that with the increase in the concentration and incubation time of the cells with CuO NPs, the H_2_O_2_ production and oxidative stress increase. So that, the antioxidant enzyme defense system loses the ability to deal with the created oxidative conditions and the activity of the CAT, APX and GPX is suppressed. In these circumstances, the suppression of enzymatic antioxidant activity, can occur due to the oxidation of proteins by ROS^58^, as well as the inactivation of enzymes by direct binding of NPs to side groups of amino acids^59^.

The effects of NPs on inducing the expression of plant secondary metabolites biosynthetic pathway genes have been reported in several studies. For example, the induction of the expression of genes in the BIAs biosynthesis pathway (*TYDC*, *DBOX*, *BBE*, and *DIOX2*) was observed in *P. somniferum* cell suspension culture treated with Ag NPs^60^. Additionally, the induction of gene expression related to the biosynthesis of phenols and alkaloids was reported in *Catharanthus roseus* callus treated with MWCNTs^61^. Furthermore, an increase in the expression of genes associated with the biosynthesis of phenols and flavonoids was noted in hairy root cultures of *Brassica rapa* treated with Ag NPs^62^. Moreover, silicon dioxide nanoparticles (SiO_2_ NPs) exhibited a remarkable effect on the induction of genes in the rosmarinic acid pathway in the hairy roots of *Dracocephalum kotschyi*^63^. In contrast to these reports, some research reported the suppression of artemisinin biosynthesis genes expression in *Artemisia annua* cell suspension culture treated with CuO NPs^64^.

Our findings in this research show that different treatments of CuO NPs used in *P. orientale* cell suspension culture medium have different effects, ranging from increasing the expression to suppressing the expression of genes at the transcriptional level, on the expression pattern of BIAs pathway genes. The use of low concentration levels of CuO NPs (20 mg/L) and increasing the treatment time (up to 48 h) in *P. orientale* cell suspension had the most effective role on increasing the expression of more studied genes (*SalSyn*, *SalR*, *SalAT*, *COR* and *CODM*).

Several studies indicate that when exposed to NPs, plant cells exhibit a diverse, complex, and interconnected array of responses across various molecular and biochemical levels. Some of these responses manifest early on, such as the onset of oxidative stress, while others arise through the transmission of stress-induced signals, leading to alterations in the expression patterns of protection-related secondary metabolites genes and quantitative and qualitative changes in metabolites^27,28^. Based on our research findings and previous studies on the impact of NPs in inducing responses leading to the production of secondary metabolites (Fig. 2.), we can infer that CuO NPs trigger the H_2_O_2_ explosion and oxidative stress. Subsequently, cells employ enzymatic antioxidant mechanisms (intensification of the activity of CAT, APX and GPX) to counteract the detrimental effects resulting from the elevated H_2_O_2_ levels (Fig. 6). Although H_2_O_2_ plays an important role as a key signaling molecule and is involved in the production of secondary metabolites in plant cells^65^, its precise role in signal transmission to downstream signaling pathways, alterations in the reprogramming of responsible gene expression, and ultimately, the production of secondary metabolites remains incompletely understood^66^. Nevertheless, the impact of ROS on the transmission of signals induced by NPs to certain downstream pathways has been determined to some extent. It appears that the signal transmission process to downstream pathways is linked to the impact of ROS on Ca^2+^ burst and increasing the activity of mitogen-activated protein kinas (MAPK)^28^. To bolster this hypothesis, prior studies underscore the elevation in Ca^2+^ levels and signaling pathway proteins in the roots of *Oryza sativa* L. treated with silver nanoparticles^67^. Furthermore, it has been discovered that the MAPK cascades and the activation of downstream transcription factors play a pivotal role in transcriptional reprogramming secondary metabolite genes^68^. Therefore, it seems that the minimum concentration of CuO NPs used (20 mg/L) induces controllable oxidative stress in *P. orientale* cells (not much H_2_O_2_ production and moderate activity of CAT, APX and GPX) (Fig. 6) and the generated H_2_O_2_ molecules plays a crucial role in initiating downstream pathways, which in turn causes the activation of defense mechanisms, such as the induction of genes for biosynthesis of secondary metabolites. Prolonging the treatment duration (up to 48 h) amplifies the duration of signal transmission, consequently augmenting gene transcript accumulation (Fig. 7). So, it can be said that both CuO NPs concentration and the duration of treatment are important in increasing the accumulation of transcripts of the studied genes.

In more severe conditions, with an increase in both the concentration of CuO NPs and the treatment time (40 mg/L CuO NPs and 48 h conditions), oxidative stress is greatly increased (indicated by a sharp increase in the amount of H_2_O_2_). Simultaneously, a reduction in the antioxidant defense function of the cell (manifested by decreased activity of CAT, APX and GPX) seems to render the cell incapable of maintaining stable conditions. Consequently, the control mechanisms for gene expression can be affected, leading to a severe decrease in the amount of transcript of the studied genes (Fig. 7). Indeed, nanoparticles are recognized as potential toxic agents for plant cells due to excessive generation of ROS and direct interaction with cell components in a dose-dependent manner causing structural damage in organelles and cells^69^. Therefore, using nanoparticles as elicitors in tissue culture conditions should be approached with high sensitivity and the optimum concentrations and the duration of treatment of cells with them should be standardized for each NPs.

The research conducted on the excitability of the *PsWRKY* transcription factor showed that the expression of this gene is affected by stimuli such as methyl jasmonate, cold, salinity and mechanical stress^26^. According to the function of the *PsWRKY* in binding to the regulatory regions of *TYDC*, it seems that a wide range of stimuli can influence the control of the biosynthesis pathway of BIAs. According to the results of our research, it was found that the expression of the *PsWRKY* transcription factor increases significantly under the treatment of CuO NPs. Therefore, this hypothesis is likely that this transcription factor is a main and upstream factor in response to a wide range of abiotic elicitors, and as an intermediate factor, *PsWRKY* plays a role in transmitting the signals from different abiotic elicitors to the responsible genes in BIAs pathway.

Increasing the amount of thebaine, codeine and morphine alkaloids in stimulated hairy roots with methyl jasmonate and salicylic acid^13^ and cell suspension culture stimulated with Ag NPs^43^ are among the first tissue culture researches of *P. orientale*. Based on the current research results, it is evident that CuO NPs effectively increased the amount of the mentioned alkaloids. Although many treatments did not perform well in this regard, the treatment with CuO NPs with a concentration of 20 mg/L at 48 h demonstrated a significant impact on the generation of H2O2, the accumulation of transcripts for genes encoding enzymes associated with the biosynthesis of morphinan alkaloids, and ultimately, a better performance in the biosynthesis and accumulation of thebaine (up to 5.07 mg/g DW), codeine (up to 3.84 mg/g DW) and morphine (up to 0.317 mg/g DW). The results of this research indicate a direct relationship can be found between the frequency of transcripts of genes encoding morphinan ring enzymes and the amount of morphinan alkaloids. The increase in transcripts level of *SalAT*, *CODM*, *T6ODM* and *COR* in the treatment with a concentration of 20 mg/L at 48 h is related to the increase in the amount of thebaine, codeine and morphine. Indeed, the findings of previous studies have consistently reported that the up-regulation of *SalAT*, *CODM*, *T6ODM*, and *COR* enhances the content of morphinan alkaloids^13,70^. This reinforces the importance of these enzymes in the biosynthetic pathway and highlights their regulatory role in the production of morphinan alkaloids in *Papaver orientale.*

Based on the findings of this research, among the three examined factors, the type of NPs (green or chemical), the concentration utilized, and the treatment duration, alterations in NPs concentration levels and the duration of treatment exert a more substantial effect in inducing oxidative stress, modifying the expression pattern of BIAs genes, and influencing the biosynthesis of morphinan. It is interesting to note that while our previous research showed that at high concentrations (40, 400 and 4000 mg/L), CuO NPs synthesized by biological compounds (green CuO NPs) leave less stress and toxicity than chemical CuO NPs on plant cells^25^, our recent research shows that the use of green and chemical CuO NPs as an elicitor in low concentrations (20 to 40 mg/L) has almost a similar effect on the occurrence of oxidative stress and the induction of the expression of BIAs biosynthesis pathway genes in *P. orientale* suspension culture.

## Conclusions

Until today, plant tissue culture techniques have effectively produced valuable plant secondary metabolites through media supplemented with various elicitors and precursors. Despite the incomplete understanding about the role of NPs on the biosynthesis pathway of secondary metabolites, these nano-structured elicitors have found a special position among the elicitors. In this research, by testing different explants of *P. orientale* under the influence of different ratios of PGRs, we found that the *P. orientale* root explants under the influence of 2 mg/L NAA and 1 mg/L BAP exhibited the best performance in terms of callogenesis percentage and biomass production. In addition, our experiment provides the first evidence that in optimizing the *P. orientale* cell suspension culture conditions for the highest efficiency of morphinan alkaloids biosynthesis, the two factors of CuO NPs concentration and the incubation time of cells are far more important than the green or chemical nature of CuO NPs so that the concentration of 20 mg/Lat 48 h had the best performance in transcript accumulation of the studied genes of the BIAs pathway and the accumulation of morphinan alkaloids.

## Data Availability

The data that support the findings of this study are available within the paper. Any other supporting data are available from the corresponding author upon request.
